# Expression of cadherin 23 isoforms is not conserved: implications for a mouse model of Usher syndrome type 1D

**Published:** 2009-09-12

**Authors:** Ayala Lagziel, Nora Overlack, Steven L. Bernstein, Robert J. Morell, Uwe Wolfrum, Thomas B. Friedman

**Affiliations:** 1 Section on Human Genetics, Laboratory of Molecular Genetics, National Institute on Deafness and Other Communication Disorders, National Institutes of Health, Rockville, MD; 2 Johannes Gutenberg-University, Institute of Zoology, Department of Cell and Matrix Biology, Mainz, Germany; 3 Department of Ophthalmology, University of Maryland School of Medicine, Baltimore, MD

## Abstract

**Purpose:**

We compared cadherin 23 (*Cdh23*) mRNA and protein variants in the inner ear and retina of wild-type and mutant mice and primates to better understand the pleiotropic effects of *Cdh23* mutations, and specifically to understand the absence of retinal degeneration in *Cdh23* mutant mice.

**Methods:**

Semiquantitative real-time PCR was used to compare the level of expression of *Cdh23* alternative transcripts in the inner ear and retina of wild-type and homozygous *Cdh23^v-6J^* (*waltzer*) mice. Antibodies generated against CDH23 isoforms were used in immunohistochemistry, immunohistology, electron microscopy, and western blot analyses of mouse and primate inner ear and retina to study the distribution of these isoforms in various cellular compartments.

**Results:**

*Cdh23* mRNA alternative splice variants were temporally and spatially regulated in the inner ear and retina. In the mature mouse retina, CDH23 isoforms were broadly expressed in various cellular compartments of the photoreceptor layer. The wild-type CDH23_V3 protein isoform, which has PDZ binding motifs but neither extracellular domains nor a transmembrane domain, localized exclusively to the outer plexiform layer of the retina containing photoreceptor cell synapses and to the synaptic region of auditory and vestibular hair cells. The longest CDH23 protein isoform, CDH23_V1, appeared by western blotting to be the only one affected by the *Cdh23^v-6J^* mutation; it was expressed in the wild-type mouse inner ear, but not in the mouse retina. However, CDH23_V1 was detected in western blot analyses of monkey and human retinas.

**Conclusions:**

The time- and tissue-dependent expression patterns that we have shown for *Cdh23* alternative transcripts suggest developmental roles and tissue-specific functions for the various transcripts. Many of these isoforms continue to be expressed in *waltzer* mice. The longest CDH23 isoform (CDH23_V1), however, is not expressed in mutant mice and is necessary for normal inner ear function. The longest isoform is expressed in the retinas of primates, but not detected in the mouse retina. This species difference suggests that the mouse may not be a suitable model for studying the retinitis pigmentosa phenotype of human Usher syndrome type 1D.

## Introduction

Usher syndrome (USH) is the most common genetic disorder that affects both hearing and vision. It is categorized into three clinical subtypes based on age of onset and severity of sensorineural hearing loss, vestibular areflexia, and retinitis pigmentosa (RP). Usher syndrome type I (USH1) is the most severe clinical subtype [1] and is a genetically heterogeneous autosomal recessive disorder. There are seven USH1 loci (*USH1B*, *USH1C*, *USH1D*, *USH1E*, *USH1F*, *USH1G*, and *USH1H*), and the causative genes for five of the loci have been identified [2-4]. The USH1 proteins myosin VIIa (*USH1B*), harmonin (*USH1C*), cadherin 23 (*USH1D*), protocadherin 15 (*USH1F*), and sans (*USH1G*) interact with each other in the inner ear and retina [5-7].

In humans, some cadherin 23 (*CDH23*) missense mutations cause nonsyndromic deafness (DFNB12), whereas truncating nonsense, frameshift, and splice site mutations have been reported to cause USH1D [8-13]. Most of the reported mutations of mouse *Cdh23* cause the *waltzer* phenotype, which is deafness and vestibular dysfunction but no retinal degeneration. *Waltzer* mice are therefore models of DFNB12 nonsyndromic deafness and not USH1D even though at least 11 of the 12 mutant alleles of *Cdh23* are hypothesized to be functional null alleles and are caused by nonsense (*Cdh23^v-3J^*, *Cdh23^v-5J^*, *Cdh23^v-6J^*), frameshift (*Cdh23^v^*, *Cdh23^v-J^*, *Cdh23^v-4J^*, *Cdh23^v-7J^, Cdh23^v-Alb^*), or splice site (*Cdh23^v-2J^, Cdh23^v-ngt^, Cdh23^v-bus^*) mutations [14-16]. One missense mutation, *Cdh23^sals^*, appears to be a hypomorph [17]. Although there are mouse models for retinal degeneration [18], thus far all waltzer mice do not develop RP and are therefore not models for USH1D [19]. The same is true for mouse mutants of the other USH1 genes including *Myo7a*, *Pcdh15*, *Sans,* and *harmonin.* Even those mutant alleles, reported to be nulls, have lacked significant retinal phenotypes [20-24]. An exception is the *Ush2a* null mouse, which develops progressive photoreceptor degeneration and moderate nonprogressive hearing loss akin to human *USH2A* patients [25].

**Figure 1:**
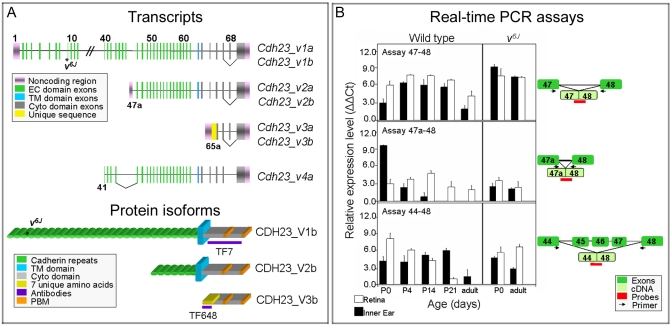
Isoforms, antisera, and expression of cadherin 23 isoforms. **A**: This panel illustrates schematically (not drawn to scale) four classes of *Cdh23* transcripts (GeneID 22295), CDH23 protein isoforms, and the locations of TaqMan probes. Gene and protein variants were designated according to Jax. Transcripts that include exon 68 are designated with an “a,” and transcripts lacking exon 68 are designated “b.” Protein variants V1, V2, and V3 are encoded by transcripts *v1*, *v2*, *v3*, respectively with (a) or without (b) exon 68. **B**: Bar graphs show data from real-time PCR assays of *Cdh23* transcripts in wild-type and *waltzer Cdh23^v-6J^* mouse inner ear (black bars) and retina (white bars) during development. The relative expression levels of *Cdh23* transcripts detected with assays 47–48, 47a-48 and 44–48 (**A**) are shown in ∆∆C_t_ values. Expression levels of *Cdh23* transcripts are reported as ∆∆C_t_ values, in which the RNA level is: 1) expressed in terms of the cycle at which exponentially accumulating cDNA product can be detected above background in an RT_PCR reaction (the threshold cycle or “C_t_”); 2) normalized to the C_t_ of *Gapdh* as an endogenous control (the “ΔC_t_”); and then 3) reported relative to an arbitrarily chosen calibrator, in this case E16.5 inner ear expression level using probe 44–48 (“∆∆C_t_”). Abbreviations: extracellular (EC), transmembrane (TM), cytoplasmic (Cyto), PDZ binding motif (PBM).

The longest *Cdh23* transcript (*Cdh23_v1a*) comprises 69 exons encoding 3,354 amino acid residues of a single pass transmembrane protein with 27 extracellular cadherin repeats (ECs). Originally, two *Cdh23* splice isoforms were reported that differed with respect to the presence or absence of exon 68, which encodes a portion of the cytoplasmic domain [8,9,11]. The CDH23 isoform, lacking the 35 residues encoded by exon 68 (*Cdh23_v1b*), is predominantly expressed in the retina [26]. The CDH23 cytoplasmic domain has two putative PDZ binding motifs (PBM; Figure 1A). These PBMs of CDH23 appear to interact with the PDZ domain-containing USH1C protein, harmonin [26], and with the scaffold protein MAGI-1, a member of the membrane-associated guanylate kinase protein family, that was shown to bind to the C-terminal PBM of CDH23 [27].

Additional shorter *Cdh23* transcripts were identified and designated isoform b (*Cdh23_v2*) and isoform c (*Cdh23_v3*). *Cdh23_v2* encodes a protein with only seven EC domains, and *Cdh23_v3* encodes a protein that lacks the EC and transmembrane domains [28,29]. Unlike *Cdh23_v1a*, which includes exon 68 and is not expressed in the retina [26], transcripts *Cdh23_v2a* and *Cdh23_v3a* are expressed in the retina [28].

In the mouse retina, CDH23 was shown to localize to the inner segment and to the synaptic terminal of photoreceptor cells in the outer plexiform layer [7,30]. In the inner ear, CDH23 was observed to localize to the transient stereocilia lateral links as well as the kinocilial links of the developing sensory hair bundle [28,29,31]. In the mature mouse inner ear, CDH23 expression was detected and was reported by us to be associated with centrosomes, kinocilial links, and Reissner’s membrane [28,32]. CDH23 is also a component of the tip link complex [33-37] together with the tip link antigen [38] identified by us as protocadherin 15 [39]. The tip link connects the tips of the shorter stereocilia to the side of its taller neighbor and gates the mechanotransduction channels located on the tops of stereocilia in all but the tallest row [40]. Recently, Rzadzinska and Steel [41] have shown that in the *Cdh23^v-2J^ waltzer* mice, tip links are present in stereocilia bundles of young hair cells, calling into question the role of cadherin 23 as a component of the tip link and suggesting that the molecular composition of the tip link is not yet fully resolved. However, the small amounts of normally processed *Cdh23* transcript (approximately 4%) reported in the *Cdh23^v-2J^* mice [14] may be sufficient wild-type expression to explain the formation of tip links in homozygous *Cdh23^v-2J^* mice.

There is a range of *Cdh23* transcripts that results from alternate promoter usage (GenBank AY563163, AY563164, AY563159, AY563160) or alternate splicing of cassette exons (GenBank AK039126). The spatiotemporal studies of CDH23 expression thus far can only partially distinguish among the various protein isoforms. In this study we investigate the expression of cadherin 23 mRNA transcripts and protein isoforms to better understand their function in the retina and inner ear, the two tissues affected in USH1D [1]. We also compare the expression of CDH23 protein isoforms in the mouse retina and inner ear as well as human and monkey retinas, in an attempt to gain better insight as to why *Cdh23* mutant mice do not develop RP, unlike humans homozygous for some mutations of *CDH23*.

## Methods

### RNA isolation and cDNA synthesis

Total RNA was isolated from 14 to 20 inner ears and retinas of wild-type (B10.A-H2^h4^/(4R)SgDvEg×C57BL/6)F1 of embryonic day 16.5 (E16.5) and post natal day 0 (P0), P4, P7, P14, P21 as well as RNA from pooled P30 to P90 inner ears and retinas. Total RNA was also isolated from P0 and adult homozygous *v^6J^* (B10.A-H2^h4^/(4R)SgDvEg×C57BL/6)F1 mice. Mice were housed in the NIDCD 5 Research Court, animal facility, and were fed NIH 07 ad libitum, and triple filtered chlorinated water was provided. Light cycles are set to be 12 h of light followed by 12 h of darkness. Animals were euthanized by a combination of CO_2_ inhalation, cervical dislocation and decapitation. All animal procedures were approved and conducted according to the NIH Animal Care and Use Committee guidelines and Animal Protocol 1263–06 to T.B.F. RNA was extracted using TRIzol^®^ (Invitrogen, Carlsbad, CA) followed by poly(A)^+^ RNA isolation using Oligotex^®^ mRNA spin-columns (Qiagen, Valencia, CA). First strand cDNA was synthesized from 1 µg of poly(A)^+^ RNA using SuperScript II Reverse Transcriptase and oligo dT priming (Clontech, Palo Alto, CA).

### Semiquantitative real-time PCR

TaqMan assays were designed using Assay by Design (Applied Biosystems, Foster City, CA). Probes hybridized to the junctions of *Cdh23* exons 47–48 (NM_023370), the junction of exons 47a-48 (GenBank AY563163, AY563164), and the junction of exons 44–48 (GenBank AK039126; Table 1) are illustrated in Figure 1A. Semiquantitative real-time PCR was performed in triplicates on an ABI 7500 real-time PCR system (Applied Biosystems). PCR reactions were performed in a 50 µl volume containing 1.5 µl cDNA, 25 µl TaqMan Universal PCR Master Mix (Applied Biosystems), 2 µl target primers and probe mix (Applied Biosystems), 1 µl control primers and probe mix (Applied Biosystems) and 20.5 µl of distilled water. Cycling conditions were 50 °C for 2 min, 95 °C for 10 min, followed by 40 cycles of 95 °C for 15 s, and 60 °C for 1 min. A probe to *Gapdh* (Applied Biosystems) was used as the endogenous control. All ΔC_t_ values are expressed relative to that of E16.5 inner ear cDNA sample using probe 44–48.

**Table 1:** Primers and probes design for real-time TaqMan assays of *Cdh23* transcripts.

**Assay 47–48**	**Sequence (5′-3′)**
Forward primer	CCAGGAAGACGCCTTTGCT
Reverse primer	CCAGCTCGCGGTTCAGA
Taqman probe	CAGACCCTGTATTGATATTC
**Assay 47a-48**	
Forward primer	GAGGAGGCGGCCACTC
Reverse primer	CCAGCTCGCGGTTCAGA
Taqman probe	TTCACCATCACAGACCCCT
**Assay 44–48**	
Forward primer	GCCGAGGTGATGGAGGACT
Reverse primer	CCAGCTCGCGGTTCAGA
Taqman probe	CTCCTGCTGGGTCTGTG

Probes were designed to flank the junctions of *Cdh23* exons 47–48, 47a-48, and 44–48.

### Statistical analysis

The data were analyzed by a two-way ANOVA test using GraphPad Prism software version 5 for Windows (GraphPad Software, La Jolla, CA). P values <0.05 were considered statistically significant.

### Expression construct

A cDNA encoding the complete sequence of *Cdh23* variant *Cdh23_v3a* (Figure 1A; GenBank AY563159) was PCR amplified from mouse P5 inner ear cDNA [39] using LA Taq DNA Polymerase (Takara Mirus, Madison, WI), cloned into the XhoI and EcoRI sites of GFP-N2 vector (Clontech) and sequence verified. Primers used for cloning: forward (XhoI): 5'-CCG GAT CTC GAG CCT ATG CAG CCC TGC TGA AGG TTC T-3'; reverse (EcoRI): 5'-CCG GAT GAA TTC CAG CTC CGT GAT TTC CAG AGG GC-3'.

### Antibodies

Antisera TF7 was generated against the cadherin 23 cytoplasmic domain (residues 2973–3215 RefSeq NM_023370) [28]. The peptide sequence CMLLPNYRAN derived from the seven unique N-terminus amino acids in CDH23_V3 (residues 1–7; MLLPNYR GenBank AY563159; Figure 1A) with the exception of the N-terminus cysteine (Princeton BioMolecules, Langhorne, PA) was used by Covance (Denver, PA) to immunize three New Zealand white rabbits producing antibodies TF647, TF648, and TF649. We purified antisera using an AminoLink Plus Immobilization Kit (Pierce, Rockford, IL). Antiserum 1D1 was generated against an epitope in the cytoplasmic domain of human CDH23 as previously described [42]. A mouse monoclonal antibody to centrin isoforms Cen1p-Cen4p was used as a molecular marker for the connecting cilium and the adjacent centriole of photoreceptor cells [43]. SNAP25 antibody was used as a marker for hair cell synaptic region (Sternberger Monoclonals Inc., Lutherville, MD). PCDH15 antiserum PB303 was raised in rabbit against a synthetic peptide (CGAEPHRHPKGILRHVKNLAELEK; corresponding to residues 1860–1882 of the mouse PCDH15 sequence, GenBank AAG53891) as previously described by Ahmed et al. [44]. Since PCDH15 was shown to be expressed in the retina, antiserum PB303 was used as a positive control in our retinal western blot analyses.

### Transfection of GFP-Cdh23_v3a into HeLa cells

We evaluated the specificity of our antibodies raised against CDH23_V3 (Figure 1A) by performing colocalization assays. Lipofectamine 2000 (Invitrogen) was used to transfect a *GFP*-*Cdh23_v3a* expression vector into HeLa cells (American Type Culture Collection, Manassas, VA), which do not endogenously express CDH23. After growth for 24 h at 37 °C in 5% CO_2_ in Dulbecco’s Modified eagle medium (DMEM; Invitrogen) supplemented with 10% fetal calf serum (FCS; Invitrogen), transfected cells were fixed with 4% paraformaldehyde and processed for immunostaining [45] using a 1:200 dilution (roughly 0.5 mg/ml stock) of antibodies TF648 and TF649. Anti-GFP antibody (Sigma-Aldrich, St. Louis MO) was used in western blots of protein extracts of transfected and untransfected HeLa cells.

### Western blot analyses

Western blot analyses were performed as previously described [28,44]. Fresh P0 to P90 inner ears and retinas of wild-type C57BL/6J and homozygous *Cdh23^v-6J^* mice were dissected. Proteins were extracted and denatured by boiling for 5 min in SDS–PAGE sample buffer (0.125 M Tris–HCl, 20% glycerol, 4% SDS, 0.005% bromo-phenol blue). A 20 µg protein sample was separated on 4%–20% Tris-glycine gels (Invitrogen) for blots incubated with antibody TF648. We used 3%–8% Tris-acetate gels (Invitrogen) for blots incubated with antibody TF7. SeeBlue Plus2 pre-stained standard (Invitrogen) was used as size markers. Gels were transferred to polyvinyldifluoride (PVDF) membranes (Millipore, Billerica, MA), and blocked overnight with 5% dry milk in TBST (10mM Tris-HCl pH 7.5, 150 mM NaCl, 0.05% Tween-20). Primary antibodies were diluted 1:1,000 from a roughly 0.5 mg/ml stock. Alkaline phosphatase-conjugated anti-rabbit secondary antibody was used at a 1:10,000 dilution (Promega, Madison, WI). ECL Plex Cy5 and Cy3 secondary antibodies were used in a 1:2,500 dilution. Fluorescent signals were detected and images captured on a Typhoon Trio Plus imaging system (GE Healthcare Bio-Science Corp., Piscataway, NJ). As a control to be sure that large retinal proteins were transferred to the membrane used in this western blot, we used validated antisera PB303 generated against PCDH15 [44] that, as expected, recognized a band of approximately 220 kDa (data not shown).

### Immunohistology of mouse retina

Some eyes of adult wild-type mice were cryofixed in melting isopentane without chemical prefixation and cryosectioned as described elsewhere [46]. Cryosections were placed on poly-L-lysine-precoated coverslips and subsequently incubated with 0.01% Tween-20 in phosphate buffered saline (PBS, Quality Biologic, Inc., Gaithersburg, MD; 90 g/l NaCl, 1.44 g/l KH_2_PO_4_, 7.95 g/l Na_2_HPO_4_ anhydrous pH 7.4 [10×]) for 20 min. After PBS washing steps (2×5 min at room temperature-RT), sections were, sections were covered with blocking solution that contained 0.5% cold-water fish gelatin plus 0.1% ovalbumin in PBS. Next, sections were incubated for a minimum of 30 min followed by overnight incubation with primary antibodies, diluted in blocking solution at 4 °C. Washed cryosections were incubated for a minimum of 1.5 h at room temperature in the dark with secondary antibodies conjugated to Alexa 488 or Alexa 568 (Invitrogen, Karlsruhe, Germany) in PBS with 4’,6-Diamidin-2’-phenylindoldihydrochlorid (DAPI; Sigma-Aldrich, Deisenhofen, Germany) to stain the DNA of the cell nuclei. After 3x10 min washings with PBS at RT, sections were mounted in Mowiol 4.88 (Hoechst, Frankfurt, Germany). Mounted retina cryosections were analyzed by microscopy (DMRB; Leica microsystems, Bensheim, Germany). Images were obtained with an ORCA-ER camera (Hamamatsu, Herrsching, Germany) and processed with Adobe Photoshop CS (Adobe Systems, San Jose, CA).

### Immunofluorescence of mouse inner ear

Mouse inner ears were removed by dissection and cochleae were perfused through the round window with 4% paraformaldehyde (PFA) and incubated in PFA for 2 h at RT. The organ of Corti and vestibular tissues were dissected from the cochlear spiral in PBS. Samples were permeabilized in 0.5% Triton X-100 (Acros Organics, Morris Plains, NJ) for 30 min and then washed in PBS. Non-specific binding sites were blocked using 5% normal goat serum (Gibco-Invitrogen) and 2% BSA (BSA, Gibco-Invitrogen) in PBS for 1 h. Samples were incubated for 2 h in primary antibodies at a concentration of ~0.5 μg/µl in blocking solution and rinsed in PBS. Samples were then incubated for 30 min in a 1:200 dilution of anti-rabbit IgG fluorescein 488 (Amersham Biosciences, Piscataway, NJ) and Alexa Fluor 594 goat anti-rabbit (Molecular Probes-Invitrogen Eugene, OR) secondary antibodies. Rhodamine-phalloidin and Alexa fluor 633 (Molecular Probes-Invotrogen) were diluted 1:100 and used to visualize F-actin. Samples were mounted using ProLong Antifade Kit (Molecular Probes-Invitrogen). Images were captured on a Carl Zeiss LSM-510 laser scanning confocal microscope.

### Immunoelectron microscopy

For immunoelectron microscopy a recently introduced protocol was applied [47]. Eyes were perforated and lenses were removed during fixation of isolated mouse eyes in 4% PFA in Soerensen buffer that contained 0.1 M disodiumhydrogenphophate and 0.1 M potassiumdihydrogenphosphate, pH 7.3. After washing (4×15 min at RT) retinas were dissected from eye cups and infiltrated with 10% and 20% sucrose in Soerensen buffer, followed by incubation in buffered 30% sucrose overnight. After four cycles of freezing in liquid nitrogen and thawing at 37 °C retinas were washed in PBS and embedded in buffered 2% agar (Sigma-Aldrich). Agar blocks were sectioned with a vibratome (Leica, Wetzlar, Germany) in 50 µm slices. Vibratome sections were blocked in 10% normal goat serum, 1% BSA in PBS and subsequently incubated with primary antibodies against CDH23 (TF7) for four days at 4 °C. After washing with PBS (4×15 min at RT) the appropriate biotinylated secondary antibodies (Vector Laboratories, Burlingame, CA) were applied to the sections. After washing with PBS (4×15 min at RT) the appropriate were visualized by a Vectastain ABC-Kit (Vector Laboratories) according to manufactor’s protocol. Subsequently, stained retinas fixed in 2.5% glutaraldehyde in 0.1 M cacodylate buffer (pH 7.3) and diaminobenzidine precipitates were silver enhanced [48,49], followed by postfixation in 0.5% OsO_4_ in 0.1 M cacodylate buffer (pH 7.3) on ice. Dehydrated specimens were embedded in araldite. Ultrathin sections (< 60 nm) were analyzed in a Tecnai 12 BioTwin transmission electron microscope (FEI, Eindhoven, The Netherlands).

### Immunohistology of human and monkey retinas

Eyes from three-year-old monkeys were obtained by S.L.B. from John Cogan (Bureau of Biologics, FDA, Bethesda, MD). Tissue was fixed in 4% PFA-PBS and frozen in OCT. Frozen sections of 10 µm were blocked for 1 h with 5% normal goat serum containing 2% bovine serum albumen. Tissue sections were reacted overnight at 4 °C with primary antibody (TF7) at a concentration of 5 µg/ml containing 2% BSA. Treated sections were incubated with alkaline phosphatase secondary antibody (Vector Laboratories, Burlingame, CA). The slides were not counterstained. Human donor eyes were obtained from a Caucasian male (74 years old) who died of congestive heart failure. Human eyes were obtained 6 h post-mortem by S.L.B. from the Maryland State Anatomy Board under IRB exemption SB-019701. The anterior segment eye tissues (cornea, iris) and posterior non-retina tissues (lens, vitreous, ciliary body) were removed. Proteins from human retina were extracted and processed for western analyses.

## Results

### *Cdh23* expression in wild-type and waltzer inner ear and retina

Three semiquantitative real-time PCR assays that could distinguish among some but not all of the *Cdh23* isoforms were used to examine the expression of *Cdh23* transcripts. Real-time PCR assay 47–48 was designed to detect a class of *Cdh23* transcripts, which includes, but is not limited to, full-length *Cdh23_v1* (Figure 1A). Expression of this class of transcripts varied significantly both by age and tissue source (Figure 1B, top panel). Two way ANOVA reveals that the expression of this class of transcripts varies significantly both by age and between tissue types, with a p-value <0.0001 for interaction of age and tissue type. Transcripts with the junction of exons 47–48 were present at all ages in both tissues, but were most abundant around age P14. The sustained expression detected in the adult inner ear was consistent with previous published reports describing expression of the longest CDH23 isoform in wild-type mouse inner ear, which appeared to be a component of the tip link [32,37]. The sustained expression may also be due to the expression of still unknown *Cdh23* splice variants.

In the homozygous mutant *Cdh23^v-6J^* inner ear and retina, the transcripts detected by assays 47–48 maintained a stable level of expression throughout development. Age and tissue have no significant effect on transcript expression levels in *Cdh23^v-6J^* mice (two-way ANOVA, p=0.05 and p=0.08 respectively). However, compared to the corresponding wild-type transcripts expression levels, the *Cdh23^v-6J^* mutation contributed to a higher level of expression in the adult inner ear and retina (two-way ANOVA; p<0.0001). The *waltzer Cdh23^v-6J^* allele is a G to T transversion occurring in exon 9 and was predicted to truncate the protein in EC3 of the longest *Cdh23* transcript *Cdh23_v1* [14,15]. Because this nonsense mutation is close to the 5′ end of *Cdh23*, transcripts that include exons 47 and 48, but are initiated from promoters downstream of exon 9, would be unaffected and detected in real-time PCR assay 47–48.

Real-time TaqMan assay 47a-48 detects transcripts *Cdh23_v2a* and *Cdh23_v2b* that contain exons 47a and 48 (Figure 1A). These transcripts use an alternate promoter upstream of exon 47a and they lack 20 ECs encoded by exons 2–46 (RefSeq NM_023370) [28]. In wild-type mice these transcripts were expressed in the retina at all time points and only transiently in the inner ear (two-way ANOVA for tissue and age; p<0.0001; Figure 1B). Expression level in the retina was unchanged in the homozygous *Cdh23^v-6J^* mutant, and was upregulated in the adult inner ear of *Cdh23^v-6J^* mutants (Figure 1B).

Assay 44–48 recognizes the class of transcripts with exon 44 spliced directly to exon 48, which we designated as isoform *Cdh23*_*v4 (*Figure 1A). The existence of *Cdh23_v4* was based on a partial cDNA (GenBank AK039126), whose promoter and protein products are unknown. There may be many different cDNAs that omit exons 45–47, so the transcript in Figure 1A was meant to represent a class of potential alternatively spliced transcripts of *Cdh23* that can be identified by our real-time PCR assay.

The expression pattern of *Cdh23_v4* is almost the converse of *Cdh23_v2* in that the expression was maximal at earlier stages in the wild-type retina and was downregulated in adult retina, while moderate expression was observed at all time points in the wild-type inner ear (Figure 1B, lower panel). As with the other assays, transcripts with this structure were detected in both the inner ear and retina of *Cdh23^v-6J^* mutant mice and were upregulated in the retina relative to the wild-type adult retina (two-way ANOVA; p<0.0001).

### Western blot analyses using CDH23_V3 antibodies

**Figure 2:**
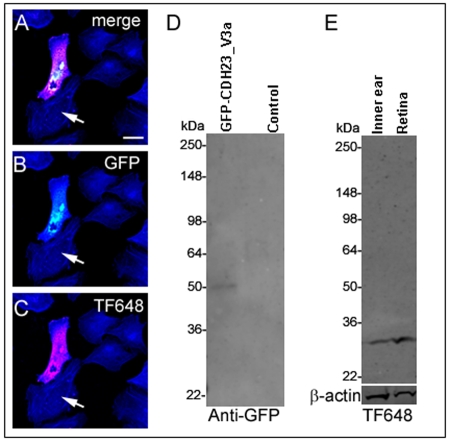
Validation of CDH23_V3 antisera. In **A-C**, arrows indicate untransfected cells; F-actin is stained in blue. **A**: A merged image showing HeLa cells 24 h after transfection with the expression vector *GFP*-*Cdh23_v3a* (see isoform designation Figure 1A). Cells were stained for CDH23_V3 with antibody TF648 (red) and F-actin antibody (blue). GFP fluorescence of transfected cells (green; **B**) overlapped with TF648 antibody staining (red; **C**) as revealed in the merged image (**A**). Scale bar equals 20 µm. **D**: A western blot was used to analyze protein extracts from HeLa cells transfected with *GFP-Cdh23_v3a* and untransfected controls. In the lysate of transfected cells a band of the expected size of roughly 50 kDa of the GFP-CDH23_V3a fusion protein (GFP approximately 27 kDa; CDH23_V3a approximately 26 kDa) was detected. **E**: Western blot of protein extracts from P60 mouse inner ear and retina revealed a band of roughly 26 kDa corresponding in size to CDH23_V3. β-actin was used as a loading control. Size standards are given in kDa.

*Cdh23_v3a* and *Cdh23_v3b* are short transcripts (708 and 600 nucleotides, respectively) initiated from a promoter in intron 65 and encode 235 and 199 amino acid proteins lacking ECs and transmembrane domains. CDH23_V3a/b expression was evaluated with antibodies TF648 and TF649, directed to the seven unique amino acids encoded at the N-terminus that differentiate this isoform from other CDH23 isoforms (Figure 1A). The antibodies were validated by transfecting a *GFP-Cdh23_v3a* (Figure 1A) expression vector into HeLa cells. Twenty-four hours after transfection the staining pattern obtained with the GFP-CDH23_V3a overlapped with the staining pattern obtained with antibody TF648. Nontransfected cells did not show antibody staining (Figure 2A-C).

Western blot analyses of transfected and untransfected cells revealed a band of approximately 50 kDa corresponding in size to the predicted size of the GFP-CDH23_V3a fusion protein (approximately 53 kDa; GFP approximately 27 kDa and CDH23_V3a approximately 26 kDa; Figure 2D). Western blot analyses of mouse inner ear and retina protein extracts revealed that antibody TF648 recognizes a band corresponding to the predicted size of CDH23_V3 (roughly 26 kDa) both in the inner ear and the retina (Figure 2E). Similar results were obtained with antibody TF649 (data not shown).

### Photoreceptor subcellular distribution of cadherin 23 isoforms

**Figure 3:**
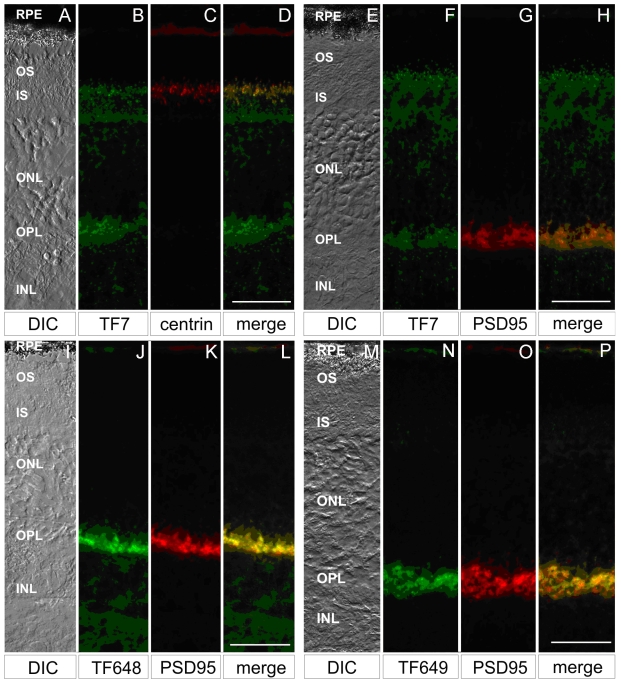
Different CDH23 protein isoforms localize to distinct cell layers of the retina. **A, E, I**, and **M** are differential interference contrast (DIC) images of longitudinal cryosections (**B-D, F-H, J-L** and **N-P**, respectively) through adult mouse retina showing photoreceptors cell layers. Indirect immunofluorescence labeling of CDH23 with the cytoplasmic domain antibody TF7 (**B**) revealed CDH23 in the ciliary region of photoreceptor cells, the ONL, and in the OPL. Double labeling with antibodies TF7 and centrin, a ciliary marker (**C**), revealed colocalization of CDH23 and centrin in the ciliary apparatus of the photoreceptor cells (**D**). **F-H**: Double labeling with antisera TF7 (**F**) and the synaptic protein PSD-95 with antisera PSD95 (**G**) revealed CDH23 colocalization with PSD-95 in the synaptic terminals in the OPL of photoreceptors cells (**H**). Double labeling with CDH23_V3 specific antibodies TF648 and TF649 (**J, N**) and PSD95 (**K, O**) revealed that CDH23_V3 was detected only in the OPL where it colocalized with PSD-95 (**L, P**). Scale bars represent 10 µm. Abbreviations: retinal pigment epithelium (RPE), outer segment (OS), inner segment (IS), outer nuclear layer (ONL), outer plexiform layer (OPL), and inner nuclear layer (INL).

**Figure 4:**
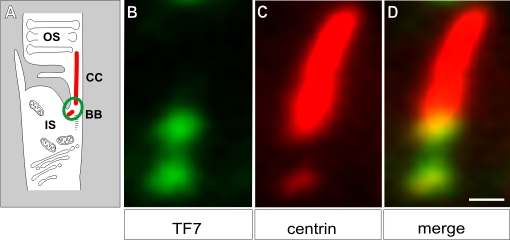
CDH23 is localized in the basal body and ciliary apparatus of photoreceptor cells. **A:** A schematic representation of a part of rod photoreceptor cell: the outer segment (OS) is linked by the ciliary apparatus composed of the connecting cilium (CC) and the basal body complex (BB) to the inner segment (IS). **B-D**: Immunofluorescence microscopy analyses of double labeling for CDH23 with TF7 (**B**) and anti-centrin (**C**) antibodies (marker for the ciliary apparatus: CC and BB) is shown in longitudinal cryosections through the ciliary part of mouse photoreceptor cells. **D**: Merged image of (**B**) and (**C**) indicates partial colocalization of CDH23 with centrin in the BB. Scale bar represents 0.25 µm.

**Figure 5:**
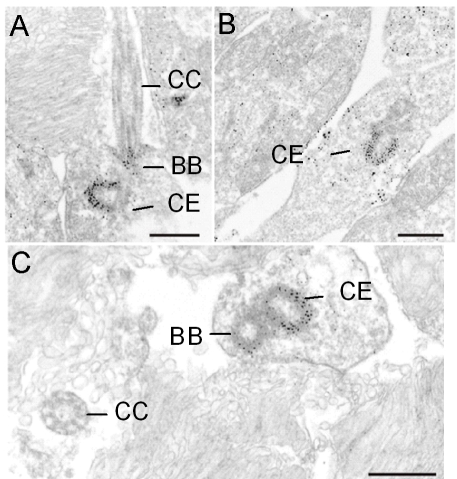
Immunoelectron microscopic localization of CDH23 in mouse rod photoreceptor cells. Electron micrographs show CDH23 labeling with TF7 antibody in ultrathin sections through parts of mouse photoreceptor cells. **A-C**: CDH23 labeling is detected in the basal body (BB) and centriole (CE) of the apical inner segment of photoreceptor cells. **A, C**: The connecting cilium (CC) is not labeled. Scale bar represents 0.2 µm.

The subcellular distribution of CDH23 in the retina was determined by analyzing cryosections through adult mice eyes by immunofluorescence with antibody TF7, which recognizes the CDH23 cytoplasmic domain, and therefore multiple Cdh23 isoforms (Figure 1A, lower panel). Cryosections of mouse retinas were double-labeled either with anti-centrin as a marker for the ciliary apparatus of photoreceptor cells [43], or anti- PSD95 as a marker for the pre- and post-synaptic terminals of photoreceptor cells located in the outer plexiform layer [50]. Double labeling with TF7 and anti-centrin (Figure 3A-D) revealed CDH23 in the ciliary region, the outer nuclear layer, and in the outer plexiform layer. Double-labeling with TF7 and anti-PSD95 (Figure 3E-H) confirmed localization of CDH23 in the outer plexiform layer. Higher resolution imaging of double-labeling immunofluorescence with TF7 and anti-centrin revealed partial colocalization of CDH23 protein isoforms and centrins in the basal bodies of the ciliary apparatus of photoreceptor cells (Figure 4). Pre-embedding high resolution immunoelectron microscopy, using antibody TF7, localized CDH23 in the basal body of the cilium and its adjacent centriole and confirmed CDH23 as a component of the ciliary apparatus (Figure 5) [42].

### CDH23_V3 localizes to the synaptic region of photoreceptor cells

Cryosections through adult mice eyes were double-labeled with the CDH23_V3 isoform specific antibodies TF648 and TF649, and an anti-PSD95, respectively. Immunofluorescence double-labeling showed colocalization of CDH23_V3 and PSD95 at the synaptic terminals of photoreceptor cells (Figure 3I-P). CDH23 immunoreactivity was also observed in this region using antibody TF7 directed against CDH23 cytoplasmic domain (Figure 3E-H). This overlap was expected since antibody TF7 should recognize CDH23 protein isoforms, which include the cytoplasmic domain or portions of it, such as CDH23_V3. No signal was detected in the photoreceptor inner segment and outer nuclear layer with CDH23_V3 specific antibodies TF648 and TF649 (Figure 3I-P).

### CDH23_3 is expressed in the synaptic region of auditory and vestibular hair cells

**Figure 6:**
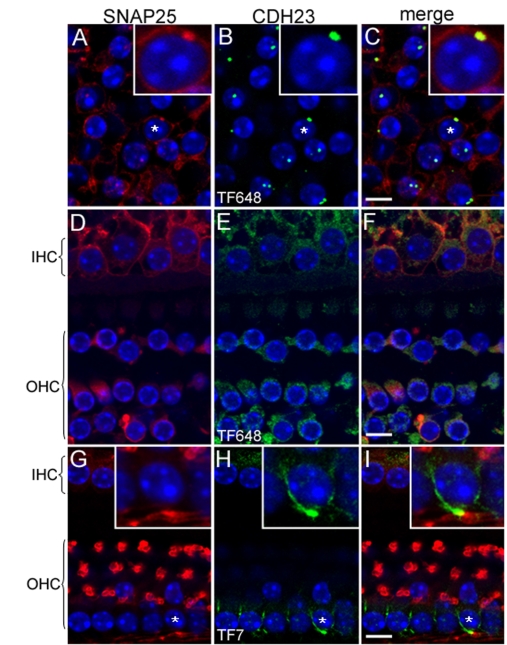
CDH23 localizes with SNAP25 to the synaptic region of mouse auditory and vestibular hair cells. In **A-I** nuclei were stained with DAPI (blue). SNAP25 is shown in red, and CDH23 in green. **A**: The synaptic region of utricular hair cells stained with SNAP25. **B**: CDH23_V3 detected with antisera TF648. **C:** In the merge image (**C**) of panels **A** and **B**, CDH23_V3 shows partial colocalization with SNAP25 in the synaptic region of utricular hair cells. The insets in **A-C** show a twofold magnification of a hair cell (asterisk) synaptic area. In the merge image (**F**) of panels **D** and **E**, CDH23_3 and SNAP25 show partial colocalization in the synaptic region of inner (IHC) and outer (OHC) hair cells. **G**: SNAP25 staining of IHC and OHC synaptic fibers. **H**: CDH23 is detected with antiserum TF7 in the synaptic fibers of IHC and OHC. **I**: In the merge image (**I**) of panels **G** and **H**, CDH23 shows partial colocalization with SNAP25 in the region of the synaptic fibers of IHC and OHC. In **G-I**, the insets are a twofold magnification of the region (asterisk), where CDH23 localizes in the synaptic fibers. Scale bars equal 5 µm.

Mouse inner ears hair cells were double-labeled with anti-SNAP25 and CDH23_V3 antibody TF648. SNAP25 localizes to the synaptic region of hair cells [51]. CDH23_V3 immunoreactivity was detected as large punctae in the basal end of vestibular hair cells, partially colocalizing with SNAP-25 in the synaptic region of vestibular (Figure 6A-C) and auditory (Figure 6D-F) hair cells. Similar results were seen with for the CDH23 cytoplasmic domain antibody TF7 and anti-SNAP25 in vestibular hair cells (data not shown) and in the synaptic fibers in the region below auditory hair cell nuclei (Figure 6G-I).

### Expression of CDH23 in the monkey retina

**Figure 7:**
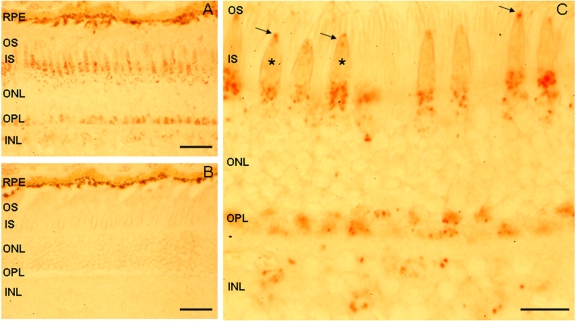
Immunolocalization of CDH23 in photoreceptor cells of monkey retina. **A**: CDH23 immunoreactivity was detected with antisera TF7 in the IS and in the synaptic terminals of the OPL of photoreceptor cells. Some staining is evident in the photoreceptor cell bodies of the ONL and INL. **B**: No immunoreactivity was observed in the controls stained only with secondary antibody. **C**: In a higher magnification, CDH23 staining is visible in the evenly spaced IS of cone-shaped photoreceptors (asterisks) and in the regions between the IS and OS where the ciliary apparatus is present (arrows). Rod photoreceptors are also positively stained (seen as thin long strings). The dark brown pigment in the upper portion of **A** and **B** is melanin of the RPE. **C:** Scale bars represent 5 µM. Magnification: **A, B** 400× and **C** 1000×. Abbreviations: retinal pigment epithelium (RPE), outer segment (OS), inner segment (IS), outer nuclear layer (ONL), outer plexiform layer (OPL), inner nuclear layer (INL).

Using CDH23 cytoplasmic domain antibody TF7, we detected CDH23 immunoreactivity in the region between the inner and outer segments of photoreceptor cells of monkey retina (where the connecting cilium and basal bodies are present), in the outer plexiform, and in the inner and outer nuclear layers, similar to the pattern observed in the mouse retina (Figure 3). Prominent CDH23 immunoreactivity was observed in the base of the cone inner segments (Figure 7A, C), a pattern of staining not observed in the mouse retina (Figure 3). Some CDH23 immunoreactivity was also detected in rod photoreceptors although less prominent than in the cone photoreceptors, possibly due to their structure and because cone photoreceptor staining is more obvious in frozen sections of monkey retina.

### CDH23_V1 is expressed in primate retina but not in mouse retina

**Figure 8:**
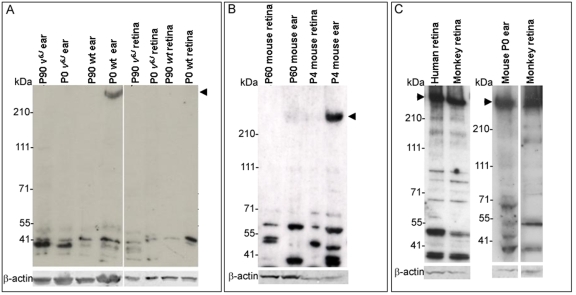
Primate and mouse CDH23 protein in inner ear and retina. **A-C**: western blot analyses of proteins separated on 3%–8% tris-acetate gels using the cytoplasmic domain antibody, TF7. **A**: Western analysis, using protein extracts from P0, and P90 wild-type and mutant mouse inner ear and retina. An approximately 350 kDa band corresponding to the largest CDH23 protein isoform was only detected in P0 mouse inner ear (arrow head). Faster migrating protein bands were present in wild-type and *Cdh23^v-6J^* and may represent lower molecular weight CDH23 protein isoforms (such as CDH23_V3). Tissue specific variation of these isoforms is better visualized in the western blot shown in panel **B**. **B**: Western analysis, using protein extracts from P4, and P60 wild-type mouse inner ear and retina. The high molecular weight band at roughly 350 kDa (arrowhead) was detected in the P4 inner ear protein sample. Traces of this band were also detected in the P60 inner ear protein sample. The faster migrating bands detected show variability in their appearance between young and adult tissue as well as variability between the inner ear and retina. **C**: Western analysis, using protein extracts from P0 mouse retina, human retina, and monkey retina. The high molecular weight band (arrow head **A-C**) detected in P0 wild-type mouse inner ear corresponds in size to the largest band detected in human and monkey retinas (arrowhead). β-actin was used as a loading control; size standards are given in kDa.

We performed western blot analyses of mouse protein extracts from P0, P4, and adult wild-type, and P0 and adult mutant inner ear and retinas using TF7 antibody. Results were obtained from 3 to 4 independent protein extractions replicated 2 to 9 times. Representative western blots (Figure 8) revealed a large band of ~350 kDa corresponding in size to CDH23 longest protein isoform (CDH23_V1) only in P0 and P4 wild-type inner ear (Figure 8A-C). This large band was not detected in wild-type P0, P4, P60, or P90 retinas and was not detected in the *waltzer Cdh23^v-6J^* inner ear or retinal samples (Figure 8A-C). A trace of this high molecular cadherin 23 isoform was detected with antibody TF7 in the P60 inner ear sample (Figure 8B, lane 2), which was consistent with the reported reduction in cadherin 23 in the mature mouse inner ear [28,29,31,32]. Various small bands were also detected both in inner ear and retina that corresponded to the predicted sizes of CDH23_V2, roughly 120 kDa or CDH23_V3, approximately 26 kDa. Some variability in the appearance of the small bands was detected between inner ear or retina, the age of the mouse and the strain, either wild-type or mutant *Cdh23^v-6J^*. A high molecular weight band of roughly 350 kDa was detected in human and monkey retinas (Figure 8C) corresponding in size to the high molecular weight band detected in P0 and P4 inner ear (Figure 8A-C).

**Figure 9:**
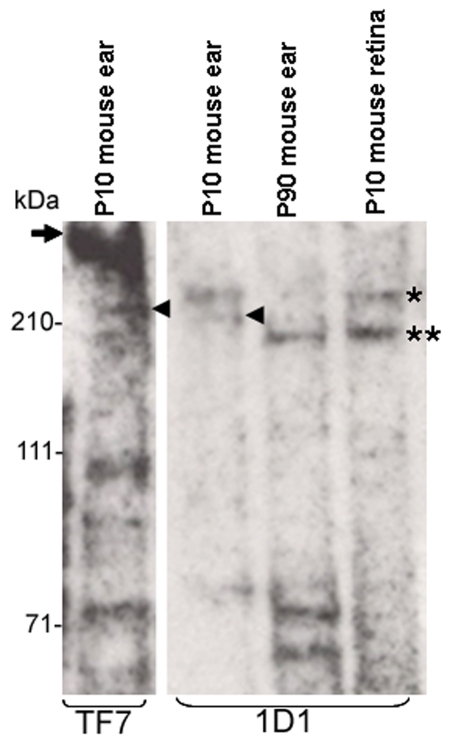
Different CDH23 antisera recognize different protein isoforms. Western blot analyses of protein extracts from P10 and P90 mouse inner ears and retinas were incubated with antisera TF7 and 1D1 generated against mouse and human CDH23 cytoplasmic domain respectively. The highest molecular weight band detected by antisera TF7 (arrow) possibly represents the longest CDH23 isoform (CDH23_V1) expressed in mouse inner ear. The same band was not detected using antisera 1D1 in the inner ear or retina. Antisera 1D1 recognized bands just above (asterisk) and below (two asterisks) the 210 kDa size marker. The band under 210 kDa was also detected by antibody TF7. An additional band (arrowhead) was detected by antisera 1D1, which seems to be detected by antisera TF7 or a band very close in size to it in the inner ear. Antisera TF7 detected a band of roughly 100 kDa that may correspond to CDH23_V2. Additional bands of lesser molecular weights were detected by both antisera, possibly representing smaller still uncharacterized CDH23 protein isoforms.

High molecular weight CDH23 detected by western analysis was previously reported in the rat, mouse, and human retina [42]. In the effort to reconcile our data with this report we performed western blot analysis of mouse inner ear and retinas using antibodies TF7 and 1D1 [42] directed against mouse and human CDH23 (respectively, Figure 9). We found that antiserum 1D1 recognized protein bands of roughly 210 kDa and roughly 70 kDa in the mouse inner ear and retina. Antibody TF7 also recognized bands of approximately 210 kDa and approximately 70 kDa, corresponding to the bands detected by antibody 1D1. But TF7 recognizes a larger band (roughly 350 kDa), not recognized by 1D1. This band may represent the longest CDH23 protein isoform with a deduced kDa of 370 that is present in the mouse inner ear at early developmental stages.

## Discussion

Most of the available localization data for CDH23 do not differentiate between the substantially different CDH23 protein isoforms [7,30,42]. We undertook this study to better characterize cadherin 23 mRNA and protein isoform expression to gain insight into their roles in the retina and inner ear. We demonstrate that *Cdh23* transcripts are developmentally regulated and have tissue-specific expression profiles. The temporal expression patterns suggest specific roles for some *Cdh23* transcripts. For example, in the inner ear, *Cdh23_v2* expression between P0 to P14 is consistent with the critical period of inner ear development. By P14, when *Cdh23_v2* is expressed at very low levels, all turns of the cochlea have reached their adult size, most vestibular sensory cells have matured, and vestibular sensory epithelia have reached their adult shape and size [52,53]. Furthermore, electrical events that lead to the onset of hearing, occurring at approximately P21, are already detectable during this period including the endocochlear potential (roughly around P12) and Preyer reflex (around P12) [54]. In the retina, *Cdh23_v4* expression between P0 to P21 is consistent with the time period in which major visual developmental events occur such as the peak of rod neurogenesis (around P0), the onset of retinal waves (approximately P1), and eye opening at approximately P13 [55,56].

In this study, we show that CDH23_V3 is expressed in the synaptic region of photoreceptor cells, and in the synaptic region of auditory and vestibular hair cells. We do not speculate on CDH23_V3 function at this location, but note that this isoform does not have extracellular or transmembrane domains. However the PDZ binding motifs remain intact, indicating it may play a part in the scaffolding network of USH proteins at the synapse [7,42].

The overall expression profile we show here for CDH23 in the outer nuclear layer, the synapse in the outer plexiform layer, and in the ciliary apparatus at the inner segment of photoreceptor cells is in agreement with previous CDH23 localization data in the retina [7,30,42]. It also overlaps with the localization of the USH proteins sans (USH1G), whirlin (USH2D), and VLGR1b, supporting CDH23 as a component of the USH retinal protein network [47,50]. In this study we show more specifically that in the ciliary apparatus, CDH23 localizes to the basal body and not to the connecting cilium. The presence of CDH23 in the basal bodies and centrioles of photoreceptors cells is in agreement with its localization in the centrosomes of auditory and vestibular hair cells. Basal bodies are analogous to centrioles that are considered to be microtubule organizers as well as the structures from which cilia extend [57,58]. Therefore, it is plausible that CDH23 plays a role in cilia development or maintenance, and in the organization of microtubules.

Our western blot analyses indicated that CDH23 protein isoform expression varies between the mouse inner ear and retina. CDH23_V1 (the longest CDH23 protein isoform) was only detected at P0-P10 in the wild-type inner ear. It was not detected in the adult wild-type inner ear or in the young and adult homozygous *Cdh23^v-6J^* mutant inner ear. The lack of CDH23_V1 expression in homozygous *Cdh23^v-6J^* mutant mice inner ear is likely to be the cause for deafness and circling behavior of mutant mice, presumably due to the disruption of hair cell bundle architecture and perhaps the tip link.

CDH23_V1 was also not detected in wild-type or *Cdh23^v-6J^* mouse retina at any time point. This observation is inconsistent with a previous report of western analysis demonstrating CDH23_V1 in mouse, rat, and human retinas [42]. We were successful in detecting CDH23_V1 in mature human and primate retinas perhaps due to a higher expression level of CDH23_V1 in these tissues. It is possible that the low abundance of CDH23_V1 in mature hair cells and in the mouse retina together with reduced sensitivity of our antibodies made it difficult for us to detect CDH23_V1. Nevertheless, human and mouse retinas appeared to express different CDH23 protein isoforms. Consistent with our data, it is plausible that CDH23_V1 is not expressed or is expressed at low levels in the mouse retina. Williams et al. [59] reported that the largest harmonin isoforms were not expressed in the mouse retina. The absence of CDH23_V1 raises the question of whether a mouse model for USH1D is possible.

Localization studies of CDH23 in the monkey retina indicate expression of CDH23 in the ciliary region, the outer plexiform layer, and in the outer and inner nuclear layer of photoreceptor cells as observed in the mouse retina. Unlike in the mouse photoreceptor cells, CDH23 is localized in the base of the inner segments of monkey cone photoreceptor cells. Considering that cytoskeletal-dependent mechanisms occur in the inner segment of the photoreceptor cells [60], it is possible that CDH23_V1 expression in the inner segment of the primate photoreceptor cells is a representation of the fundamental differences that exist between the human and mouse vision processes [61,62].

In conclusion, we show here that CDH23 protein isoforms are localized in discrete compartments of the photoreceptor layer of the retina and the inner ear. We also show that CDH23 has several wild-type isoforms shorter than CDH23_V1, and demonstrate that there are differences in CDH23 isoform expression between mice and primates. If one of the short isoforms of CDH23 in the mouse retina performs the function of the long isoform of CDH23 in primates, then studying the expression and function of these shorter wild-type isoforms in the mouse retina may lead to a creation of a mouse model for USH1D. A knockout model of CDH23 cytoplasmic domain shared by the majority of CDH23 isoforms would test this hypothesis.
